# The current applications of nano and biomaterials in drug delivery of dental implant

**DOI:** 10.1186/s12903-024-03911-9

**Published:** 2024-01-24

**Authors:** Lotfollah Kamali Hakim, Amir Yari, Nariman Nikparto, Saeed Hasani Mehraban, Sahar Cheperli, Amirali Asadi, Amirmohammad Arabi Darehdor, Sayna Nezaminia, Dorara Dortaj, Yasin Nazari, Mohamad Dehghan, Pardis Hojjat, Mahsa Mohajeri, Mohammad Saleh Hasani Jebelli

**Affiliations:** 1 Oral and Maxillofacial Surgeon, Tehran, Iran; 2 Department of Oral and Maxillofacial Surgery, School of Dentistry, Kashan University of Medical Sciences, Kashan, Iran; 3 Oral and Maxillofacial Surgeon (OMFS), Department of Oral and Maxillofacial Surgery, Masters in Public Health (MPH), Zanjan University of Medical Sciences, Zanjan, Iran; 4 Department of Oral and Maxillofacial Surgery, School of Dentistry, Tehran University of Medical Sciences, Tehran, Iran; 5 Periodontist and Implantologist, Tehran, Iran; 6 Oral and Maxillofacial Surgeon, Department of Oral and Maxillofacial Surgery, School of Dentistry, Tehran University of Medical Sciences, Tehran, Iran; 7 Operative Department, School of Dentistry, Tehran University of Medical Sciences, Tehran, Iran; 8 General Dentist, Masters in Engineering, Tehran, Iran; 9 Specialist in Prosthodontics, Independent Researcher, Tehran, Iran; 10 Department of Periodontics, Faculty of Dentistry, Zahedan University of Medical Sciences, Zahedan, Iran; 11 Department of Prosthodontics, School of Dentistry, Tehran University of Medical Sciences, Tehran, Iran; 12 Periodontology Department, School of Dentistry, Tehran Medical Science Islamic Azad University, Tehran, Iran; 13 Oral and Maxillofacial Surgeon, School of Dentistry, Alborz University of Medical Sciences, Tehran, Iran; 14 Oral and Maxillofacial Surgery Resident, Department of Oral and Maxillofacial Surgery, School of Dentistry, Tehran University of Medical Sciences, Tehran, Iran

**Keywords:** Nanomaterials, Biomaterials, Drug delivery, Dental implant

## Abstract

**Background and aim:**

Dental implantology has revolutionized oral rehabilitation, offering a sophisticated solution for restoring missing teeth. Despite advancements, issues like infection, inflammation, and osseointegration persist. Nano and biomaterials, with their unique properties, present promising opportunities for enhancing dental implant therapies by improving drug delivery systems. This review discussed the current applications of nano and biomaterials in drug delivery for dental implants.

**Method:**

A literature review examined recent studies and advancements in nano and biomaterials for drug delivery in dental implantology. Various materials, including nanoparticles, biocompatible polymers, and bioactive coatings, were reviewed for their efficacy in controlled drug release, antimicrobial properties, and promotion of osseointegration.

**Results:**

Nano and biomaterials exhibit considerable potential in improving drug delivery for dental implants. Nanostructured drug carriers demonstrate enhanced therapeutic efficacy, sustained release profiles, and improved biocompatibility. Furthermore, bioactive coatings contribute to better osseointegration and reduced risks of infections.

**Conclusion:**

Integrating current nano and biomaterials in drug delivery for dental implants holds promise for advancing clinical outcomes. Enhanced drug delivery systems can mitigate complications associated with dental implant procedures, offering improved infection control, reduced inflammation, and optimized osseointegration.

## Introduction

In regenerative medicine, considerable progress has been made in improving patients’ quality of life and life expectancy. The development of biomedical implants has contributed significantly to modern healthcare. Metal and non-metal implants with permanent or temporary biomaterials are common in orthopedics, maxillofacial and cranial surgery, ophthalmology, and neurosurgery. Biomaterials can generally form bacterial adhesions and biofilms regardless of their anatomical location, and biomaterials represent one group of materials that can develop these problems [1–4].

By delivering local drugs directly to tissues, unnecessary antimicrobials can be reduced. In addition to hydrogels and nanoparticles, polymers were also tested for releasing topical antibiotics [5]. The drug concentration at the implant site must also be stable and effective so that bacteria cannot become resistant. Considering that dental implants are expected to last for many decades, the drug-release coating must be able to recharge/replace itself when needed. An appropriate drug delivery agent is PDLA, even though it only improves drug release temporarily. Minocycline microspheres have been used to treat infections around implants for over 20 years [6–9]. Though it often manifests as a slow, chronic condition, it can develop early after surgery or years afterward. Without treatment, peri-implantitis can cause significant bone loss around the implant, osteomyelitis, abscesses, sinusitis, pneumonia, as well as pathological fractures of the jawbone [1]. 3D printing, called AM, can fabricate several devices. These technologies have many potential applications, but bioengineering is among the most promising. Research using 3D printing can simulate bones, cartilage, or heart valves closely resembling biological tissues. Using AM technology, geometric cues can be created accurately. Growing demand and application of tissue engineering, antimicrobial/anti-biofouling devices, and regenerative medicine have prompted researchers to look for new manufacturing technologies to solve the tissue and organ supply shortages and immunological requirements of implanted devices. Several fields can benefit from this technology. Artificial hips and knees, heart valves, stents, and even vascular grafts are often made from polymeric materials to improve quality of life and, in some cases, increase life expectancy [10–14]. In oral medicine, drugs are often released locally to treat oral diseases. 3D printing can also design unique drug delivery systems because of its precision and three-dimensional composition. The chlorhexidine coating on mouthguards inhibits bacteria growth in the mouth. A wearable oral delivery device constructed with FDM mouthguards containing preloaded drugs. A 3D-printed mouthguard delivers drugs efficiently in personalized dental therapeutics [15, 16]. Drug-eluting implants prevent infections associated with dental implants and orthopedic implants. Metallic implants are often coated with polymer or ceramic to embed drugs. A metallic implant surface can also be incorporated with covalent bonds, self-assembled layers, and silver nanoparticles. The polymer-based layers are suspected to cause complications in addition to loosening from the implant site, chemical changes, and corrosion. For this reason, inorganic coatings are being studied as a drug delivery system. Not much attention has been paid to metallic drug-eluting systems. The purpose of this mini-review is to summarize recent advancements in implant drug delivery systems [6, 17, 18]. Dental implants are sealed with healing abutments, which are permanent permucosal implants. This stage increases the risk of infection and failure. Bacteria can be reduced around biomaterials to enhance healing. As a result of the simple design and structure of healing abutments, temporary local drug delivery devices can easily be modified and used as temporary local drug delivery devices. Bacterial infiltration during implant healing is reduced by preventing infection and poor implant-tissue interfaces due to bacterial leaks [1, 19–22]. Nanotechnology has gained significance in dentistry for its potential to enhance materials’ properties at the nanoscale. Nanomaterials can be tailored to interact with biological systems and provide targeted delivery. Biomaterials in Dental Implants: Biomaterials play a crucial role in the success of dental implants, providing the necessary strength, biocompatibility, and integration with the surrounding tissues. Nano-based drug delivery systems can offer localized and sustained release of therapeutic agents. This can be particularly beneficial in the oral environment, providing controlled release directly at the implant site. Nanoformulations allow for precise control over drug release kinetics, improving the therapeutic efficacy of drugs while minimizing side effects. Nano coatings on implant surfaces can help prevent microbial adhesion and reduce the risk of infections. Nanostructured coatings on implant surfaces may enhance osseointegration by promoting better interaction with surrounding bone tissues. Biomaterials can be engineered to deliver anti-inflammatory drugs to mitigate inflammation associated with the implantation process. Ensuring the biocompatibility and long-term safety of nano and biomaterials in the oral environment is a key challenge. Moving from experimental studies to practical clinical applications requires addressing regulatory and translational hurdles. Highlight recent studies showcasing specific nanoparticle formulations that have demonstrated success in drug delivery for dental implants. Discuss any ongoing or recently completed clinical trials investigating the application of nano and biomaterials in drug delivery for dental implants. The integration of nano and biomaterials in drug delivery for dental implants holds great promise for improving the success and longevity of dental implant procedures. Ongoing research continues to explore new materials, formulations, and delivery strategies to address challenges and pave the way for clinical implementation [1, 2, 23–29]. In this review article, the current applications of nano and biomaterials are discussed in drug delivery of dental implants.

## Method

A literature search was conducted to identify relevant clinical studies on nano and biomaterials in dental implants. Databases such as PubMed, Scopus, and Web of Science were systematically queried using keywords including “nanomaterials,” “biomaterials,” “drug delivery,” and “dental implant.” The search was limited to articles published in English. Studies were included if they focused on using nano and biomaterials for drug delivery in the context of dental implants.

## Biomaterials in drug delivery of dental implant

Dental and oral diseases demonstrated the potential of biocompatible materials and systems. Any organ, tissue, or body function can be improved or replaced by a biomaterial that conducts electrically from a system that directly contacts biological tissues. Dental research studies hyaluronic acid, gelatin, collagen, and chitosan due to their capabilities as native tissues [30–32]. Maxillofacial surgery and dentistry use CP in different ways. Several bone graft and dental implant methods have failed because of external infectious diseases and microbial biofilms. Infectious procedure percentages can be reduced, and situations can be improved when antibiotics and CP are combined. For antibiotic delivery, CP and doped vehicles with appropriate mechanical and physicochemical properties are needed [33]. As a result of pyrophosphate hydrolysis in physiological environments, calcium pyrophosphate, metaphosphate, and orthophosphates are formed. The CP matrix may also contain antibiotics distributed in controlled release as prevention or treatment [4, 34, 35]. Thus, most drug-eluted ceramic nanoscaffolds are multifunctional, such as delivering drugs, encouraging cell growth, and directing tissue regeneration. There is no doubt that ceramic scaffolds provide superior mechanical support than polymeric scaffolds. The surface area, grain size, and calcium-to-phosphorus ratio of calcium phosphate nanoparticles can further be tailored to control drug-release kinetics. Self-templating molecules have been used to fabricate well-controlled hollow silica nanospheres. Silica nanospheres with hollow cores can accumulate eight times more drugs than their solid counterparts, according to studies. These hollow silica nanospheres also allowed for time-delayed multiple-stage releases [28, 36].

Research has demonstrated the significant roles played by various growth factors, including BMPs, PDGF, IGFs, and VEGF in craniofacial growth [37]. Smad-dependent signaling and MAPK signaling are activated during skeletal development and bone formation. Wound healing, bone repair, and remodeling are all affected by fibroblast growth factor signaling during trauma or infection [38]. Skeletal growth and maintenance are supported by IGFs, similar to IGFs. Also, VEGF influences proliferating, vascularizing, and ossifying of the maxillary and palatine mesenchyme as well as calvarial ossification. These growth factors may be beneficial in enhancing healing in patients with severe craniofacial anomalies due to an imbalance in these factors [39]. To elicit specific biological responses in humans, growth factors require high doses and multiple injections because of their short half-life in circulation, limited diffusion, rapid degradation, and cleavage [40]. ECM molecules safeguard and stabilize growth factors in vivo. To achieve localized and sustained release of growth factors, an appropriate carrier system must be selected [41]. Many materials have been designed to entrap growth factors within or on substrates, including sponges, nanofibrous membranes, micro/nanoparticles, and hydrogels [42]. BMP-2 and VEGF delivered simultaneously in rats almost completely repaired size defects [43, 44]. Different methods of immobilization can be used to control growth factor release [45]. Preclinical and clinical studies of these delivery systems remain limited despite extensive in vitro studies. rhBMP-based products are commercially available. Using rhBMP-2 embedded in an absorbable collagen sponge, sinus lifts and localized alveolar ridge augmentations can be achieved [46, 47]. Several carrier-based grafts are approved for clinical use, including OP-1 Putty, a collagen graft infused with rhBMP-7 [48]. Growth factor-containing materials not only maintain controlled release kinetics of growth factors but also provide a porous osteoconductive framework for bone ingrowth. Combining or sequentially delivering multiple growth factors can also accelerate bone regeneration. Despite the challenges associated with determining the right concentrations of growth factor combinations, customizing release profiles, controlling gradients, and timing, various delivery vehicles have proven effective in stimulating bone healing and angiogenesis [49–51]. For instance, combinations of PDGF/IGF-I in methylcellulose gels have shown increased defect filling in periodontal lesions during phase I/II human clinical trials [52, 53]. In recent years, approaches have been developed that incorporate biomimicry of bone and the surrounding soft tissues of the peri-implant surrounding the implant. Several extracellular matrix proteins, peptides, and growth factors have been used to modify dental implant surfaces. Using these biofunctional coatings, osseointegration and peri-implant soft tissue integration can be enhanced and maintained, reducing the risks of biofilm-induced peri-implant inflammation. Bioabsorbable polymeric coatings on titanium surfaces can release osteoconductive or antibacterial molecules over time. Wet and submerged simulated body fluids with calcium and phosphorus have coated titanium surfaces with HA [54–56]. Calcium-phosphorus coatings, including HA, are essentially osteoconductive to bone. Calcium/phosphorus ratios, crystallinities, and coating thickness are all factors that affect the biodegradation properties of these materials. HA is commonly coated on Ti implant surfaces using plasma spraying (a conventional atmospheric plasma-spraying method). A spray’s chemical and physical properties are affected by the spray’s parameters, such as its flame combination and spraying flow rate [23, 29]. After five years, there has been an assessment of approximately 95% clinical success with HA-coated implants. The success rate of implants has now dropped significantly to under 80% after 10 years. HA coating layer problems may have caused such a low success rate. Clinical evaluations of cylindrical implants were conducted, however. Clinical trials are nevertheless needed to evaluate calcium–phosphorus coatings in greater detail [13, 29, 57]. Implants restore oral function and aesthetics by replacing missing teeth. Materials like titanium and its alloys are often used in these implants. There has been a revolution in tooth replacement thanks to dental implants, which are very successful. Osseointegration occurs when an implant fuses with surrounding bones due to mechanical properties. To improve long-term effectiveness and aesthetic outcomes, new implant designs, surface modifications, and implant-abutment connections are being investigated [24, 58–60]. Porous HA can be manufactured through ceramic slip foaming, replicated reticulated foam scaffolds, destruction of sacrificial porogens like polymer beads, or hydrothermal conversion of calcium-based coral or bone. Drug delivery systems can be developed with spherical porous HA granules. Water and sodium chloride were adjusted to adjust the structure of spherical granules with various pore and channel structures. The release of anti-inflammatory or antibacterial drugs from HA at implantation sites was studied in a previous study. Several drugs have been found to enhance bone formation at the implant site so that HA can be loaded with these agents. It is being investigated whether the complex microchannel structures of HA granules can be used to control the release rate of drugs [28, 58, 61, 62].

On the surface of the coating, osteoblasts attach directly to the surface of the HA coating, demonstrating its biocompatibility with hard tissue. It has been reported that metal implants coated with HA enhance bone apposition and prevent metal-ion release into the bone. However, there are a few critical issues with the HA coating layer. There are several reasons for the failure of Ti dental implants. HA particles that have delaminated or worn out impede the healing process of the bone and cause inflammation around the implant. An implant in a load-bearing area is prone to breaking because of the thick coating layer. Coating calcium-phosphorus with different coating techniques has been successfully achieved and investigated recently. Other calcium-phosphorus coatings, however, do not offer the same long-term clinical benefits as plasma-sprayed HA [29, 63–65]. The similarity between HA and natural bone mineral makes it an ideal bone graft and implant material. Implants in orthopedic and dental applications undergo osteointegration to integrate with the surrounding bone. It promotes bone cell attachment and growth for reconstructive procedures, joint replacements, and dental implants. HA is used in tissue engineering as a scaffold material for tissue regeneration. Porous structures allow cells to infiltrate, travel nutrients, and develop new tissue. The HA scaffold provides mechanical support and guides tissue growth by mimicking the natural extracellular matrix. These implants repair cartilage, regenerate bone and manufacture tissues. Integrating HA with 3D printing and additive manufacturing has led to a new era of personalized implants [24, 63, 66, 67].

TiO_2_ nanotubes with HA-enhanced bone tissue integration. TiO_2_ nanotubes were modified with carbon nanotubes, polymers, and proteins. Matrix-assisted pulsed laser evaporation (MAPLE) can deposit polymeric materials and heat-sensitive biomolecules. Osteogenic cells can be stimulated by coating HA or titanium with bioabsorbable molecules. Recent approaches include using peptides and peptidomimetics as titanium surface additives. Biomolecules have been used to enhance bone healing because of their chemical and functional versatility. Adsorption, entrapment, and covalent binding are the three main methods of immobilizing molecules. Collagen in the extracellular matrix of titanium dental implants enhances osseointegration and soft tissue growth, thus improving the seal between the implant and the gum. Cells can also be attached to the extracellular matrix by coating titanium with osteopontin and bone sialoprotein. Though large extracellular matrix proteins are low in chemical stability and quickly resorbable in biological fluids, they may still be helpful [54, 68]. Recently, HA carriers have attracted considerable attention as drug delivery systems. Several biomedical applications can benefit from these systems, including controlled and targeted drug release [24].

In most cases, HA coatings allow metallic implants to become more biocompatible and osseointegrated, reducing the risk of implant failure and improving their stability over the long term. It often mimics natural bone minerals using HA and other bioactive coatings. These coatings can stimulate bone formation and healing around implants. Dental implants have been found to integrate better with bioactive coatings, particularly in compromised clinical settings. Researchers seek to develop multifunctional coatings, optimize coating techniques, and research novel coating materials. Electrochemical deposition, plasma spraying, and biomimetic mineralization are used to optimize the composition and adhesion of HA coatings. Implants used in orthopedics, dentistry, and other biomedical fields are improving in durability and performance due to advancements in coating methodologies. In terms of biocompatibility and safety, HA is one of the most advantageous drug carriers. The body tolerates it well since it is a natural component of bone. Low cytotoxicity, minimal inflammatory response, and good biocompatibility have been demonstrated for HA-based drug delivery systems. In biomedical applications, particularly in regenerative medicine, this makes them ideal [24, 69, 70]. Biological and mechanical implant properties are enhanced through approaches such as implant coatings in implant dentistry. Biocompatibility, antibacterial properties, and bioactivity can be enhanced with different coatings applied to zirconia surfaces. As a result of their bioactivity, bioactive coatings on zirconia can induce the formation of hydroxyapatite in biological environments, which is necessary for promoting bone growth [71].

The successful integration of a biomaterial into the host tissue and the resulting clinical outcomes are primarily influenced by the host’s immune response to the foreign biomaterial. These interactions between biomaterials and the immune system are intricate, and gaining a comprehensive understanding of them could enhance their regenerative capabilities [72]. Upon the implantation of any biomaterial, there is an immediate triggering of inflammatory reactions to safeguard the adjacent tissues. The biomaterial’s surface becomes coated with an initial layer of proteins as host plasma proteins adhere to it [73]. Fibrinogen, in particular, plays a role in attracting inflammatory cells to the implant surface, facilitating platelet adhesion, and promoting the formation of chemoattractant-rich clots for further cellular growth [74, 75]. It seems that a controlled immune response is beneficial for biocompatible biomaterials to effectively fulfill their intended functions. The nature of these immune reactions is, in part, shaped by immune cells such as mast cells, macrophages, and lymphocytes. Mast cells, for instance, release fibrosis-inducing molecules and pro-fibrogenic cytokines, which hinder tissue regeneration and promote fibrosis rather than healing [76]. M1 macrophages exhibit classical activation, whereas M2 macrophages promote wound healing [77, 78]. A prolonged presence of M1 macrophages negatively impacts bone regeneration, despite their importance in the initial stages of bone repair. Consequently, the bone regeneration process needs to transition from proinflammatory M1 to anti-inflammatory M2 activities [79]. Bone regeneration and healing are dependent on this shift between M1 and M2 phenotypes, rather than being driven by a single specific phenotype [80]. Bone replacement biomaterials, including CP biomaterials like DCP bioceramics, have provided significant benefits to orthopedic and dental patients worldwide, overcoming the limitations of natural bone grafts [81]. To achieve regenerative outcomes comparable to natural bone grafts, the performance of these biomaterials must be further enhanced. Strategies for modulating, rather than suppressing, the immune response have been explored to promote better integration and regeneration performance with implanted bone biomaterials. To achieve this, smart biomaterials have been designed to activate the desired immune response, facilitating tissue/material integration and remodeling [82]. The adhesion of immune cells and the secretion of cytokines may be directly influenced by ECM proteins. When tissue damage occurs, signaling molecules are released, activating the repair immune response by stimulating the TLR of resident immune cells [83].

An aerosol deposition method was used to evaluate the osteogenic potential of zirconia coated with HA for improved osseointegration by Cho et al. (2015). A thin layer of HA on zirconia showed shallow, regular craters as analyzed by SEM and XRD. By measuring the thickness and uniformity of the HA films by SEM, a significant improvement in the wettability of the surface coated with HA was demonstrated. Based on confocal laser scan microscopy, the attachment of MC3T3-E1 preosteoblasts to titanium and zirconia surfaces did not differ significantly; however, cells attached to the zirconia with HA showed a lower proliferation rate than cells attached to the uncoated zirconia. However, the osteogenic response to HA-coated zirconia was shown to be remarkable. The findings indicate that HA coatings promote osteogenesis and improve surface modification [84].

HA coatings with 4-Hexylresorcinol components were tested in vitro and in vivo by Kim et al. (2011). HA and 4-HR were successfully deposited onto titanium surfaces using an aerosol deposition technique, confirmed by x-ray diffraction and Fourier transform infrared. HA + 4-HR coatings were more adhesion-efficient than HA alone. Osteocalcin expression was significantly increased with the HA + 4-HR coating compared to the HA-only coating. Following eight weeks, 4 h-coated dental implants were removed faster than HA-only implants. A significant increase in bone formation and bone-to-implant contact values was observed in the HA + 4-HR group 8 weeks after surgery. Implants coated with HA + 4-HR were more durable than those coated with HA alone. It can be considered an option in tooth extraction cases or poor bone quality [85].

Lee et al. (2014) studied the growth of peri-implant bone using collagen, hydroxyapatite (HA), and collagen plus HA (CH) implants in combination with uncoated, hydroxyapatite (HA), and collagen plus HA implants. Coating of HA on titanium was observed in a characteristic phase. Diffraction patterns were maintained after collagen and BMP-2 coating, but collagen and BMP-2 were not. It was confirmed that collagen exists by infrared absorption. Bone formation around the implant and bone-in-crack were significantly enhanced by CH surfaces over UC surfaces. BMP-2 added to implant surfaces was less effective than CH coatings. CH group was significantly more likely to form new bone and have a higher BIC. There was no significant difference between the other groups [86]. Comparing different types of silica-coated micropatterned zirconia surfaces for fibroblast adherence and antibacterial effects, Laranjeira et al. (2014) According to the study results, zirconia coated with silica lowers bacterial adhesion based on surface morphology. Additionally, microstructured bioactive coatings can improve the adhesion of soft tissues, fibrin network formation, and cell growth. By reducing biofilm adherence and improving protein adsorption, they reduce biofilm formation and increase soft tissue adherence [87]. Y-TZP and HA were mixed in various ratios by Pardun et al. (2015) to produce coatings. In the experiments, osteoblast adhesion and proliferation were stimulated by dissolving HA. The bioactivity of calcium phosphate increased when immersed in simulated body fluid, but its mechanical and chemical stability decreased. Based on the author’s research, coatings with more outstanding tetragonal zirconia content have excellent interfacial bonding, mechanical strength, and bioactivity potential [26].

## Nanomaterials in drug delivery of dental implant

BDDS can be created by incorporating nanoparticles, primarily metallic nanoparticles. In vitro and in vivo studies using metallic nanoparticles emphasizing antibacterial properties have been well explored in the literature. Several dental biomaterials contain metal-based nanoparticles. Periodontal procedures demonstrate the most appropriate use of antibacterial biomaterials, bone substitutes, and membranes for tissue regeneration and drug delivery. It has been demonstrated that metallic nanoparticles effectively modify various biomaterials. The aim is either to enhance their properties or to facilitate nanoparticle release [88, 89]. A wide range of applications have been developed for nanomaterials in recent years. The properties of nanoparticles make them ideal for medical applications due to their size and physicochemical properties. These agents can enter the human body through the digestive tract, lungs, or skin. The type and nanosize range of medicines determine the type and scale of nanomedicines in nanotechnology. Using nanosized particles, nanomedicine can diagnose, prevent, treat, and improve human health and preserve it [90]. Dental implants are successful only when the hard tissues heal, and the soft tissues form and heal around them. Biological seals between soft tissues and implants minimize the risk of peri-implantitis, prevent oral bacteria from entering the body, and even make implant restoration more aesthetic [27]. A successful dental implant depends on good osseointegration. As a consequence, osseointegration enhancement has been a popular research topic. After titanium and its alloys are subjected to surface modification, surface modification by LbL electrostatic self-assembly promotes osteoblast or stem cell attachment and osteogenic differentiation and increases the expression of osteogenesis markers [27, 91, 92]. Recent years have seen a rise in the interest in nanomaterials for use in regenerative or restorative dentistry. Some of the fields that are benefited by nanomaterial technology [93].

### Gold and metallic particles

A gold nanoparticle is a new spherical nanoparticle with a dielectric core surrounded by a metallic shell. The particles’ chemical and optical properties make them ideal for therapeutic applications and biomedical imaging. The delivery of sensitive drugs, proteins, peptides, and genes is possible by using drug delivery systems containing gold nanoparticles. Nanoparticles with a size between 20 and 50 nm are highly receptive, while those with a size between 40 and 50 nm are toxic. These nanoparticles are used in dental implants, cancer diagnosis, and tissue and bone engineering. Additionally, gold nanoparticles of different shapes have been synthesized using various mechanisms [89, 94]. A gold nanoparticle is essential in detecting periodontal disease as soon as possible. A lack of periodontium-supporting tissue and alveolar bone endorses the movement of teeth in periodontal disorders [95]. To inhibit aggregation and enhance stability, high-paramagnetic metallic particles have phospholipids, dextran, or another coating. Phospholipid-, dextran-, or another anti-aggregation compound coats a superparamagnetic agent with metallic particles [90].

#### Nano-silver (nAg)

In addition to preventing biofilm formation, silver nanoparticles have antibacterial properties. Thus, these technologies are used for dental implantology, periodontology, and alveolar bone regeneration to prevent bacterial contamination and provide biocompatible scaffolds. AgNPs are embedded in a scaffold to confer bactericidal properties while maintaining their structure and properties [96]. The antimicrobial activity of AgNPs and other antibiotics in coating formulations has been favorable. To coat titanium substrates, AgNPs were combined with tantalum nitride. Against Staphylococcus aureus, the composites containing 21.4 weight% silver had significant antibacterial effects. As compared to uncoated samples, coated samples showed improved viability and proliferation. No information was provided regarding AgNP size (size not provided), keeping Candida from colonizing the implant. During implant placement, albicans grew in the microgap. In the first group, AgNP suspension was used on the internal surface of the screw (abutment); in the second group, sterile PBS was applied to the internal surface; and in the third group, AgNPs were applied. A sterile SDB suspension was not used for G1 or G2, while a sterile suspension of C. albicans was used for G3. In the positive control group, C. albicans colony-forming units were statistically higher than those in the AgNPs group. There was no contamination in the negative control group [97].

In vivo osseointegration was studied by Svensson et al. (2013). Commercially available Pt, Au, and Ag coatings were evaluated in vivo on titanium implants. A total of 16 adult rabbits were implanted with coated femurs and tibiae, and one rabbit received control implants. A 99% reduction in adhesion was observed in vitro with coated implants, compared with non-coated implants [98]. The AgNPs embedding technique has also been used by Zhu et al. (2015) to evaluate titanium disks’ antibacterial and osteogenic properties. A comparison of control and AgNPs treatments did not show significant differences in proliferation, viability, and differentiation of bone marrow stem cells. Microorganisms that colonize peri-implant tissues, Fusobacterium nucleatum and Staphylococcus aureus, were inhibited by immobilized AgNPs [99]. Flores et al. (2013) investigated titanium coatings containing citrate-capped AgNPs for their antimicrobial and biocompatibility properties. With the first approach, AgNPs were dispersed in a liquid medium for antibacterial evaluation, whereas [100] with the second approach, AgNPs were immobilized on titanium implants for antibacterial evaluation against sessile bacteria. It was observed that the mixture was bactericidal [101]. In Qiao et al. (2015), titanium implants were sandblasted and acid-etched before AgNPs were embedded. With a plasma immersion/ion implantation technique, six Labrador dogs had implants inserted into their jaws. A comparison of AgNP-embedded samples with sandblasted or acid-etched controls showed greater bone mineral density, bone formation, and trabecular pattern. To enhance osteoconductivity, hierarchical micro/nanotopography mimics the extracellular matrix structure in nature [102].

#### Titanium (Ti)

**Fig. 1:**
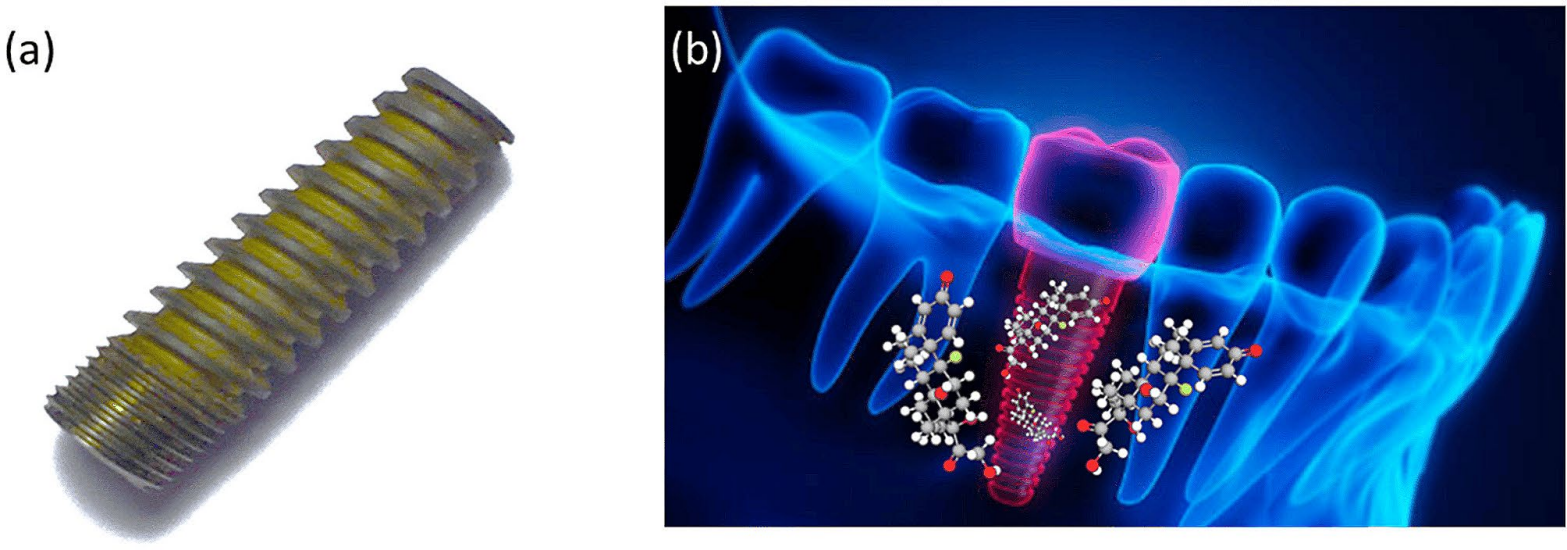
(**a**) An implant coated with DEX has been developed for titanium, and (**b**) in the implantation site, DEX is released schematically [6]

For almost half a century, titanium dental implants have had a low failure rate worldwide. Fixture osseointegration and implant-prosthetic success may be influenced by bone quality and quantity. Atrophic posterior maxilla are typically rehabilitated with dental implants [103]. Using titanium alloys in dental and orthopedic applications is becoming increasingly popular. A titanium alloy’s mechanical properties make it suitable for long-term, heavy-load applications such as hip joint replacements, bone screws, and plates. Since titanium has limited mechanical properties, it is usually used for dental implants. Cells must be able to adhere to, reproduce, and mature on the implants and provide the platform for fusing with the surrounding tissue. An implant has been osseointegrated when the surrounding bone can resist shear loads, and the distance between the implant and tissue is kept to a maximum of 50 cm to prevent fibrous capsule formation around the implant. In the case of microroughened implants, osteoblastic activity is directly directed on the surface by contact osteogenesis, which occurs after new bone fuses with existing bone tissue around the implant [104]. A modified material containing antibiotics has been developed to coat surfaces with modified materials to control biofilm formation and infection associated with implants. Recently, antibiotics have become increasingly popular in surface coatings on Ti materials. As a result of proteins adsorbing on top of these coatings, they exhibit reduced release and cytotoxicity. This treatment’s surface topography properties are also important in determining its antimicrobial activity [6]. A controlled antibiotic drug delivery system for coating metallic implant surfaces has been proposed. Controlled release rates and selective agent coatings are among the advantages of these systems. Material biological performance must be maintained or enhanced by antimicrobial surface coatings. Due to their homogeneous drug release, a monolithic system may be formed by applying antimicrobial agents to dental implants. In Fig. 1a, dexamethasone-coated Ti dental implants are shown developed. Figure 1b shows a schematic representation of the release of DEX [6].

**Fig. 2:**
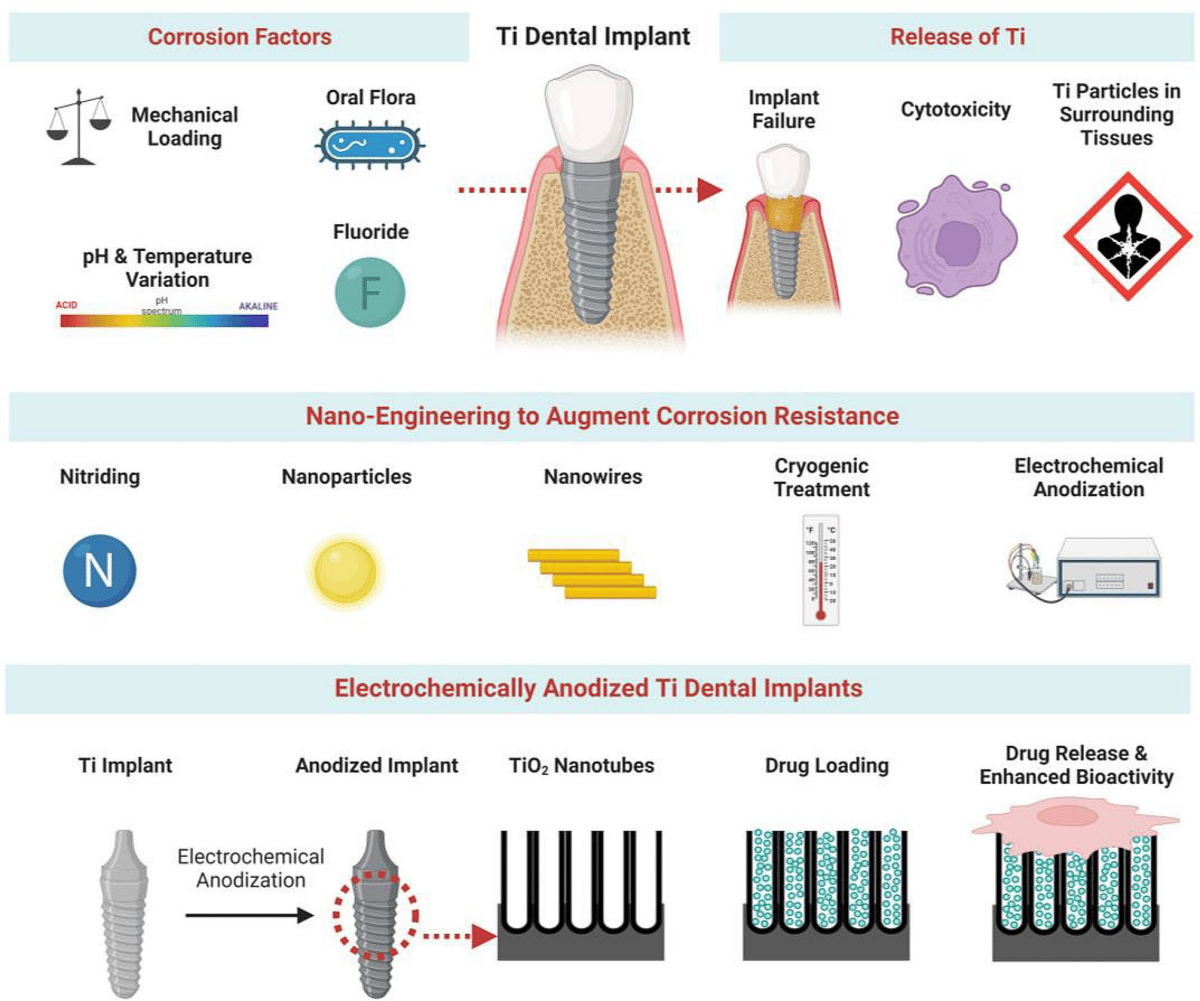
Ti dental implants corrode and deliver local drugs. The diagram shows corrosion factors, nano-engineering to improve corrosion resistance, and anodized Ti dental implants for enhanced bioactivity and drug release [105].

It has been reported that corrosion can cause Ti ions/particles to be released from Ti-based dental/orthodontic implants, compromising their ability to integrate with tissue. Several nano-engineering techniques have been reported to improve Ti implants’ corrosion resistance and reduce their particle release/ion release (Fig. 2). An overview of the mechanisms and factors contributing to the degradation of ions/particles from titanium implants, as well as their influence on surrounding cells and tissues, is presented in this comprehensive review. Additionally, the deposition of nanostructures, the refinement of grains, and electric arcing methods have been discussed in detail to enhance the corrosion resistance of Ti implants. Specifically, the review focuses on anodized Ti dental implants capable of eluting powerful therapeutics locally for enhanced implant integration [105].

Brunello et al. (2022) found that reducing bacteria colonization and improving soft tissue attachment were crucial for maintaining healthy peri-implant tissues. They used titanium nitride to coat anodized surfaces [106]. ZrN was tested against oral bacteria similarly isolated from clinical specimens to our investigations. Study results showed that TiN coatings inhibited oral bacterial growth significantly [2]. Poly (lactic-co-glycolic acid)/Silver/ZnO nanorods coated on titanium implants demonstrated excellent biocompatibility and cytocompatibility as well as long-term antibacterial activity, according to Xiang et al. (2017) [107]. To strengthen the cohesiveness of metallic fixtures with bone, Goel et al. (2019) sputtered nanocomposite Ti or zinc oxide thin films. Antibacterial activity was also demonstrated in the developed thin film [108].

#### Zinc

Trace elements such as zinc are essential for our muscles, bones, skin, and teeth health. The FDA recognizes GRAS. Its unique characteristics include optical, magnetic, mechanical, electrical, catalytic, and photochemical, making it an excellent material for dental applications. To alter the properties of the compound, researchers can adjust its size, add additional compounds, or adjust synthesis conditions. With decreasing particle size, desirable characteristics become more apparent [106, 109]. Memarzadeh et al. (2015) examined the effects of ZnO nanoparticles on dental implant surfaces to reduce the risk of failure and improve osteointegration. The underlying osteoblasts demonstrated an increased proliferative response in the presence of ZnO NPs coated on the implant surface for 5 and 10 days. As well as antimicrobial properties, they also exhibited antibacterial properties. These properties can enhance bone growth, inhibiting infection [110]. A modified titanium implant material containing ZnO nanoparticles and N-halamine was developed and demonstrated remarkable antibacterial properties by Li et al. (2017) [111]. Physicochemical, osteogenic, apatite nucleation, corrosion resistance, and osteogenic properties of ZNO biofunctionalized thin films with DMP1 peptides were improved in a 2018 study by Trino et al. [112]

### Ceramic nanoparticles

Nanoparticles such as titanium, silica, and alumina have recently emerged as drug carriers in organic systems. Due to their non-biodegradability, these compounds have undesirable effects [90]. Regenerative medicine and biomaterials engineering are rapidly finding applications for nano-hydroxyapatite. Biocompatibility with the physiological environment is a characteristic and principal constituent of dental tissues and bones. Calcium and OH groups are found in multisubstituted carbonated nHA with crystalline nanostructures. Due to its tissue-compatible and osteo-inductive properties, nHA is extensively used in bone supplements and fillers. In addition to drug delivery systems, the ability of nHA to bind to biological barriers makes it suitable for targeted and controlled DDSs. By simply injecting drugs into porous nHA, bone tissue can be treated with drugs and strengthened when new cells are formed. These systems may be utilized to develop the most advanced DDSs for treating and preventing bone diseases, including tumors, metastases, and osteoporosis. The nHA-based DDSs can deliver antibiotics directly to infected bone tissue. A bacterial infection in the bones is best treated with antibiotic therapy. By preventing secondary infections after bone fractures, prosthesis insertion, and transplant surgeries, researchers can maximize the success of these procedures. A prosthesis may degrade or separate if secondary infections are not controlled [25].

### Carbon nanomaterials

**Fig. 3:**
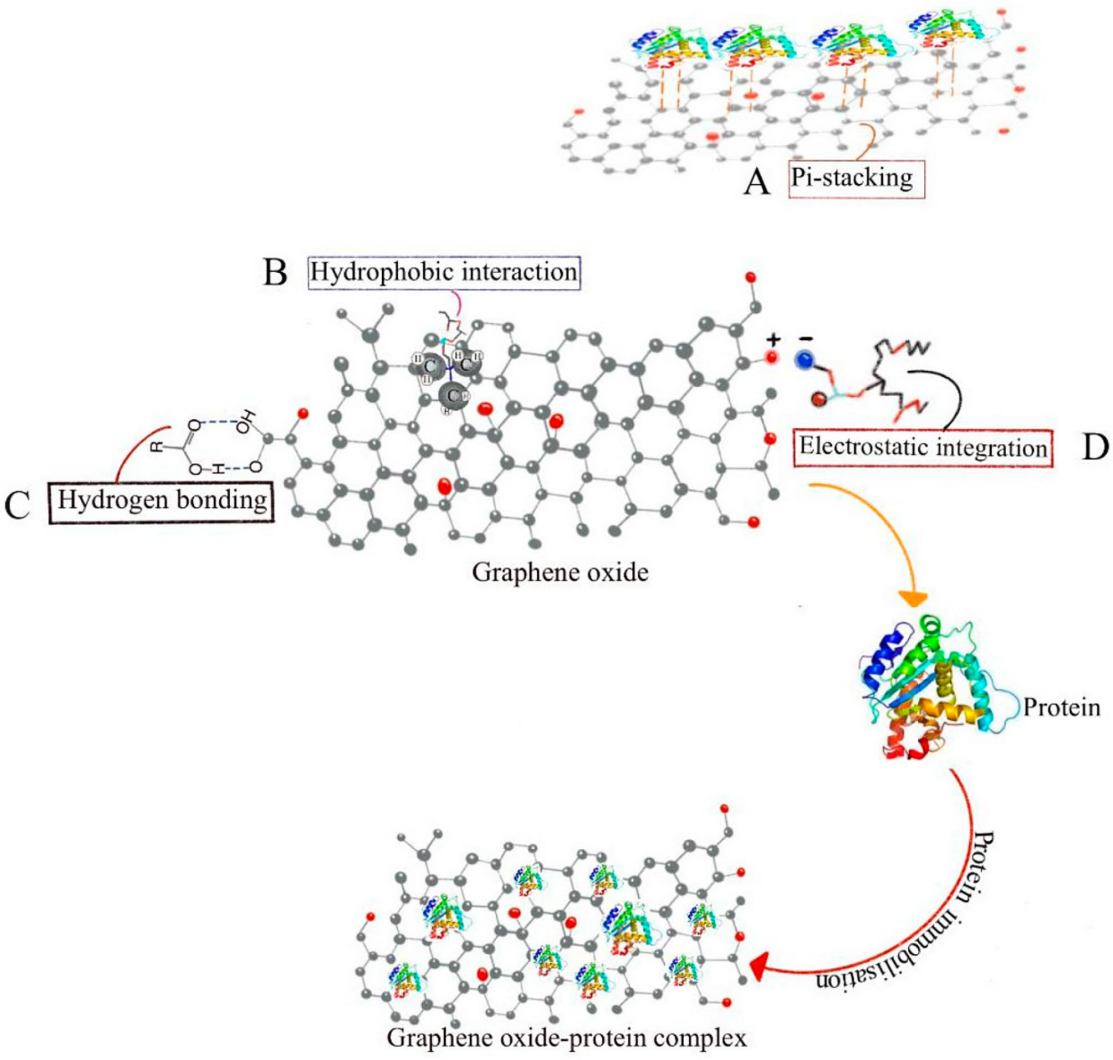
Various non-covalent bonds in protein binding are illustrated. (**A**) graphene oxide binds proteins through noncovalent pi-stacking interactions; (**B**) hydrophobic interactions enhance the bioactivity and stability of proteins; (**C**) the structure of proteins is stabilized by hydrogen bonds; (**D**) for proteins to fold, stay stable, be flexible, and function properly, electrostatic integration is vital [113]

Fullerenes and nanotubes are examples of carbon nanomaterials. The surface of a fullerene can be functionalized to bind tissues and consists of only 60 carbon atoms. Nanotubes are electrically conductible and highly strong. Graphite sheets rolled into seamless cylindrical shapes can be visualized structurally as a single sheet. Compounds with one wall and compounds with more than one wall are divided into two classes [90]. The unique properties of CNMs make them an ideal candidate for use in dentistry. Besides acting as drugs, they may also serve as gene carriers. CNMs have been combined with drugs, proteins, nucleic acids, and bioactive peptides to achieve low toxicity and anti-inflammatory properties by combining CNMs with graphene. Proteins, nucleic acids, and cells have been used for biological medication delivery using stacking interactions. Hydrogen bonds, van der Waals forces, electrostatics, hydrophobic interactions, and positive-negative interactions are noncovalent interactions that confine drug molecules (Fig. 3). A CNM coating can, therefore, serve as a drug-releasing system on biomimetic dental implants, facilitating wound healing and osseointegration after a wound has healed [113]. CNMs are established antibacterial agents and are also capable of delivering drugs, most commonly via contact-mediated biocidal actions. As a result of attracting and attracting positively charged nanoparticles, bacteria’s cell walls become more permeable, causing their membranes to rupture, and allowing intracellular organelles to leak out. Nanocomposite materials are more effective than conventional antimicrobial strategies because of their small particle size and surface-to-volume ratio [113].

## Limitations

While integrating nano and biomaterials in drug delivery for dental implants holds significant promise, it is imperative to acknowledge several limitations inherent in the current state of research and clinical applications. Biocompatibility and safety concerns: One of the primary concerns in using nano and biomaterials revolves around biocompatibility and safety. Although extensive research has been conducted to evaluate the biocompatibility of these materials, long-term effects and potential adverse reactions in the complex oral environment are still not fully elucidated. Standardization and regulatory challenges: The field of nano and biomaterials for dental implant drug delivery lacks standardized protocols and regulatory frameworks. The absence of universally accepted guidelines can hinder the reproducibility of studies and impede the seamless transition from research to clinical applications. Establishing standardized methodologies and regulatory benchmarks is essential for ensuring the reliability and safety of these innovations in diverse clinical settings. Clinical validation and long-term efficacy: While numerous in vitro and preclinical studies have demonstrated the potential efficacy of nano and biomaterials in drug delivery, the leap to clinical validation remains a significant challenge. Limited long-term clinical trials with large sample sizes are available, and their findings may not capture the intricacies of patient diversity and varying oral health conditions. Robust, well-designed clinical trials are imperative to validate these technologies’ long-term efficacy and safety in diverse patient populations. Cost implications: Incorporating nano and biomaterials into dental implant drug delivery systems may introduce additional costs in material production, manufacturing processes, and regulatory compliance. Economic considerations must be carefully addressed, including the affordability of these advanced technologies for clinicians and patients. Striking a balance between innovation and cost-effectiveness is essential to ensure widespread accessibility and adoption. Intra- and inter-individual variability: The response to nano and biomaterials can vary significantly among individuals due to genetic, lifestyle, and environmental factors. Tailoring drug delivery systems to accommodate this variability is a complex challenge. Customization based on patient-specific factors may be required for optimal therapeutic outcomes, necessitating further research into personalized approaches considering the diverse biological responses observed in the oral cavity. Limited understanding of oral microbiome dynamics: The intricate and dynamic nature of the oral microbiome adds another layer of complexity to using nano and biomaterials in drug delivery. The potential impact of these materials on the oral microbial community, including both pathogenic and beneficial microorganisms, requires further exploration. In conclusion, while integrating nano and biomaterials in dental implant drug delivery holds immense promise, addressing these limitations is essential for realizing their full potential in clinical applications. Collaborative efforts among researchers, clinicians, and regulatory bodies are crucial to overcoming these challenges and advancing the field toward safer, more effective, and widely accessible solutions for enhancing dental implant therapies.

## Future direction

The field of dental implantology has undergone remarkable advancements in recent years, with nano and biomaterials playing a pivotal role in enhancing the efficacy of drug-delivery systems associated with dental implants. As researchers stand at the intersection of materials science, nanotechnology, and dentistry, the future holds tremendous promise for further innovation and refinement in this interdisciplinary domain. Advances in precision medicine and biomaterial engineering may allow for tailoring drug release profiles based on patient-specific factors such as age, health status, and genetic predispositions. Personalized drug delivery systems have the potential to optimize therapeutic outcomes, minimize side effects, and enhance overall patient satisfaction with dental implant treatments. Integration of smart materials: Smart materials, capable of responding to specific physiological cues or external stimuli, hold great promise for the future of dental implant drug delivery. Innovative materials can enable on-demand drug release, responding to the oral environment’s pH, temperature, or bacterial presence. This real-time responsiveness could significantly improve the precision and efficiency of drug delivery, minimizing the risk of complications and ensuring optimal therapeutic effects. Nanotheranostics in implant dentistry: The convergence of nanotechnology and diagnostics, known as nanotheranostics, is anticipated to revolutionize dental implantology. Nanoparticles with both therapeutic and diagnostic functionalities could be designed to provide real-time monitoring of the implant site while simultaneously delivering therapeutic agents. This integrated approach can potentially enhance treatment outcomes by enabling early complication detection and timely intervention. Targeted drug delivery for tissue regeneration: Incorporating growth factors, stem cell therapies, or other regenerative agents into nanostructured biomaterials could facilitate precise delivery to the implant site, fostering enhanced tissue integration and minimizing the risk of peri-implant diseases. This targeted approach may pave the way for improved long-term success rates of dental implant treatments. Multifunctional coatings for implant surfaces: Advancements in surface engineering are expected to develop multifunctional coatings for dental implant surfaces. These coatings may serve as drug delivery vehicles and provide antimicrobial properties, enhance osseointegration, and modulate the inflammatory response. Integrating multiple functionalities into a single coating could streamline the implantation process and contribute to dental implants’ overall success and longevity.

## Data Availability

All data generated or analyzed during this study are included in this published article.

## Abbreviations

AM: Additive manufacturing
CP: Calcium phosphate
HA: Hydroxyapatite
BMP-2: Bone morphogenetic protein-2
FDM: Fused deposition modeling
3D: Three-dimensional (3D)
Y-TZP: Yttria-Stabilized Tetragonal Zirconia Polycrystal ceramics
LbL: Layer-by-Layer
BMPs: Bone morphogenetic proteins
PDGF: Platelet-derived growth factor
IGFs: insulin-like growth factors
VEGF: Vascular endothelial growth factor
ECM: Extracellular matrix
MAPKs: Mitogen-activated protein kinases
DCP: Dicalcium phosphate
TLR: Toll-like receptors
BDDS: Biomaterials with drug-delivery systems
nAg: Nano-silver
AgNPs: Nano-silver nanoparticles
HGFs: Human gingival fibroblasts
SDB: Sabouraud dextrose broth
PBS: Phosphate buffered saline
GRAS: General recognition of its safety
DDSs: Drug delivery systems
CNMs: Carbon nanomaterials
